# School re-entry for children and adolescents with cancer: A qualitative study from the perspectives of school personnel

**DOI:** 10.1016/j.apjon.2026.101009

**Published:** 2026-07-24

**Authors:** Birgül Erdoğan, Tuba Arpaci

**Affiliations:** a Department of Pediatric Nursing, Faculty of Health Sciences, Kocaeli University, Kocaeli, Türkiye; b Department of Pediatric Nursing, Faculty of Health Sciences, Karamanoğlu Mehmetbey University, Karaman, Türkiye

**Keywords:** Childhood cancer, School re-entry, School personnel, Qualitative research, Pediatric oncology nursing, Educational continuity

## Abstract

**Objective:**

School re-entry after a cancer-related interruption may involve academic, social, emotional, and health-related adjustment. Evidence from school personnel remains limited. This study explored school personnel's perspectives on school re-entry among children and adolescents undergoing or having completed childhood cancer treatment.

**Methods:**

This qualitative descriptive study was conducted in Türkiye with 12 school personnel recruited through purposive maximum-variation and snowball sampling: eight teachers, two school administrators, one school counselor, and one school nurse. Data were collected through online semi-structured interviews between April 2025 and January 2026 and analyzed using a team-based inductive thematic analysis informed by Braun and Clarke's six phases.

**Results:**

Three analytic domains encompassing eight themes were identified: academic and health-related adjustment; social and emotional reintegration; and school-side support needs and coordination gaps. Participants described school absence as involving academic disruption, weakened belonging, peer-related vulnerability, and a need for individualized adjustment. Support was often dependent on individual initiative, with limited school-useable health information, unclear role boundaries, and insufficient coordination.

**Conclusions:**

School personnel described school re-entry as a gradual, individualized process requiring coordinated communication, planning, and follow-up. The school-side needs identified may inform future interdisciplinary models linking children and families, schools, and health care teams. Pediatric oncology nurses may contribute to communication and planning; however, role-specific recommendations require direct investigation with nurses, children, and families.

## Introduction

Advances in the diagnosis and treatment of childhood cancer have led to improved survival rates, making it increasingly important to focus not only on children's physical recovery after treatment, but also on their reintegration into social, emotional, and academic life. In this context, return to school should be understood not merely as the resumption of education for children and adolescents who have experienced cancer, but as a process of rebuilding peer relationships, restoring a sense of belonging, coping with visible or invisible treatment-related effects, and moving toward the “normalcy” of everyday life.^1^^,^^2^ Recent systematic reviews have shown that returning to school is a multidimensional process in which children may face numerous barriers, including lack of information, academic delay, peer reactions, stigma, fatigue, cognitive difficulties, and unstructured or poorly coordinated transitions.^3^, ^4^, ^5^ For children receiving or completing cancer treatment, these difficulties are closely linked to ongoing symptom burden, psychosocial adjustment, developmental needs, and the continuity of supportive care across home, hospital, and school settings.

Readjustment to school is a multifactorial process involving not only the child and family, but also teachers, school administrators, school counselors, and school health professionals. An effective return-to-school process can be achieved through structured guidance, multidisciplinary collaboration, and the active involvement of the child.^6^ In particular, school personnel play a critical role in recognizing changes in the student's academic performance, planning classroom accommodations, informing peers, responding sensitively to visible differences or health-related needs, and maintaining coordination among the family, health care team, and school. Nevertheless, recent studies indicate that teachers and other school personnel often experience uncertainty, feelings of inadequacy, and unmet information needs when supporting the return of a student receiving treatment for or with a history of childhood cancer to the classroom.^7^^,^^8^ Similarly, recent reviews and qualitative studies focusing on parental experiences have shown that school reintegration is often hindered by the absence of systematic, coordinated, and sustainable support structures, as well as by deficiencies in information exchange, role clarification, and individualized planning.^9^, ^10^, ^11^ These findings suggest that school reintegration is not solely an educational transition, but also a care transition that requires communication, preparation, and continuity across sectors. From a pediatric oncology nursing perspective, school personnel's perspectives may be particularly relevant because they reflect the school-facing context in which cancer-related needs are interpreted and managed. School personnel are often the first to observe whether clinical advice is understandable in the classroom, whether treatment-related needs are translated into feasible school routines, and where communication between the family, hospital, and school breaks down. Understanding their perspectives may therefore help identify school-side information gaps, coordination problems, and practical concerns that could inform future interdisciplinary return-to-school support.

Different groups of school personnel may encounter different aspects of the student's return-to-school process.^6^, ^7^, ^8^ Teachers are usually the professionals who most directly observe the child's classroom participation, learning difficulties, fatigue, peer interactions, and day-to-day adjustment. School administrators may have less direct interaction with the student, but they often play a central role in communication with parents or guardians, arranging attendance procedures, coordinating institutional resources, and supporting school-wide decisions about accommodations and safety. School counselors are positioned to observe psychosocial adjustment, peer acceptance, emotional difficulties, and the classroom climate, whereas a school nurse or a nurse working in a school infirmary, where available, may be more directly involved in monitoring health-related vulnerabilities, infection precautions, medication-related needs, fatigue, and emergency responses.^6^, ^7^, ^8^^,^^12^ Therefore, examining the perspectives of school personnel in different roles is important because school re-entry is not experienced or supported through a single professional lens; rather, it is shaped by the combined academic, psychosocial, administrative, and health-related responsibilities distributed across the school environment.^6^^,^^7^

From a pediatric oncology nursing perspective, return to school may be understood as a cancer-related supportive-care issue that intersects with the child's quality of life, psychosocial well-being, family functioning, and long-term adjustment across the cancer care continuum.^12^ Current psychosocial care standards emphasize that supportive care in childhood cancer should extend beyond disease-focused boundaries and include the child's reintegration into daily life, school, and the social environment.^13^ Pediatric oncology nurses play an important role in providing holistic care, including symptom management, psychosocial support, family education, and coordination of care throughout the cancer trajectory.^14^ In addition, facilitating educational continuity and supporting school reintegration require interdisciplinary collaboration among health care professionals, families, and schools, as described in the school-reentry literature.^4^^,^^11^ Within future interdisciplinary return-to-school models, the school-side information and coordination needs described in the literature suggest potential areas such as translating clinical knowledge into practical school-related information, supporting family preparation, identifying treatment-related needs that may affect school participation, and facilitating communication between families, school personnel, and health care teams. Accordingly, potential areas of involvement for pediatric oncology nurses and school nurses or nurses working in a school infirmary may include assessing the readiness of the child and family, identifying concerns and needs related to return to school, facilitating communication with the school, supporting the information needs of school personnel, and strengthening interdisciplinary coordination.^2^^,^^12^ School personnel may also require clear and practical information about issues such as fatigue, infection risk, cognitive changes, emotional distress, medication-related needs, and visible treatment effects to support individualized planning for a safer and more responsive return-to-school process. Notably, a recent scale development study highlighted the need for tools that can be used by pediatric nurses and school nurses or nurses working in a school infirmary to systematically assess return-to-school readiness across child, family, and school contexts.^15^ However, because the present study did not directly interview pediatric oncology nurses, children, or families, its nursing relevance is not based on direct claims about what nurses should do. Rather, it is based on identifying school-side information, communication, and coordination needs that pediatric oncology nurses could consider when supporting return-to-school planning.

The Turkish context also shapes the relevance and transferability of these findings. In Türkiye, school health nursing is a developing field that has gained clearer regulatory visibility in recent years; however, school nurses are not uniformly embedded in every school.^16^^,^^17^ The Ministry of National Education issued a directive defining the working procedures and responsibilities of school health nurses, including student health monitoring, follow-up of students with special health needs, health education, infection prevention, emergency response, and coordination with school administration and families.^17^ Nevertheless, unlike settings such as Australia, where formal health support planning processes and school liaison mechanisms have been developed to facilitate communication among schools, families, and health care providers, Türkiye does not have a standardized national school liaison system specifically for children returning to school after cancer treatment.^18^ In this respect, the Turkish context also resembles settings such as Japan,^19^ where school reintegration may depend on collaboration among hospital schools, families, and local schools; however, in Türkiye, coordination is often shaped by the initiative of individual health care professionals, families, and school personnel rather than by formalized procedures. Recent Turkish literature also emphasizes that school health nursing continues to develop within the framework of current legal regulations and that awareness of school health nurses’ roles and responsibilities among school administrators remains an important issue.^16^^,^^20^ Therefore, in many school settings, communication between pediatric oncology teams and schools may occur primarily through teachers, administrators, and school counselors rather than through a school nurse or a nurse working in a school infirmary, who is consistently present on site. This makes the perspectives of non-nurse school personnel particularly important for understanding how health-related information is received, interpreted, and implemented in everyday school practice.

This context differs from, but also resonates with, other Asia–Pacific settings where school health infrastructures vary considerably. In Japan, school health activities are supported by a long-established structure that includes the Yogo teacher, a school-based professional who plays a pivotal role in first aid, health checks, health observation, infection prevention, environmental hygiene management, health counseling and guidance, school health room management, and health-related educational activities.^21^ In some Australian jurisdictions, students with health conditions are supported through formal health support planning processes that require collaboration among school staff, parents or guardians, and health professionals, with student health support plans developed and updated according to the student's medical, health, and personal care needs.^22^^,^^23^ In India, the School Health and Wellness Programme under Ayushman Bharat designates and trains two teachers in each school as Health and Wellness Ambassadors to deliver health promotion and disease prevention activities, illustrating another model in which teachers carry structured health-related responsibilities within schools.^24^^,^^25^ These examples demonstrate that school health support across the Asia–Pacific region is organized through different professional roles and institutional mechanisms. However, across these diverse systems, children with cancer returning to school require clear, school-useable health information, developmentally appropriate accommodations, and communication pathways linking families, schools, and health care teams. In this sense, exploring school personnel's perspectives in Türkiye may offer transferable insights for other settings where school-based health support is unevenly distributed or where teachers and administrators carry substantial responsibility for implementing health-related guidance.

In the existing literature, studies on the return-to-school process for children and adolescents undergoing or having completed childhood cancer treatment have focused largely on the experiences of children or parents.^11^^,^^26^, ^27^, ^28^ The situation is similar in Türkiye, where research on return to school remains limited. However, little is known about how school personnel perceive this process, what challenges they encounter, what kinds of information and support they need, and which factors facilitate or hinder readjustment. Understanding school personnel's perspectives may be relevant for pediatric oncology nursing because these perspectives can reveal school-side points at which communication may break down, school-related concerns may remain insufficiently addressed, and future nursing-informed support could be better aligned with information, preparation, and coordination needs during school reintegration. This gap is important for the development of school-based practices and for informing family-centered, interdisciplinary approaches to school reintegration in pediatric oncology care. Accordingly, this study aimed to examine the views of school personnel regarding the return-to-school process of children who had interrupted their education due to childhood cancer. More specifically, the study sought to generate knowledge that could inform potential ways in which pediatric oncology nurses may be involved in future interdisciplinary return-to-school models, particularly through family preparation, school communication, individualized guidance, and interdisciplinary coordination. By focusing on school personnel rather than on nurses themselves, the study does not claim to generate direct evidence of nursing interventions; instead, it aims to clarify the school-side needs and contextual realities that pediatric oncology nurses may need to consider when collaborating with families and schools. The study may contribute to the development of nursing-informed, family-centered, and interdisciplinary approaches that support school reintegration.

## Methods

### Study design

This study was conducted using a qualitative descriptive design to examine the experiences, perceptions, and suggestions of school personnel regarding the return-to-school process of children and adolescents who had interrupted their education due to childhood cancer during or after treatment. Qualitative description allows experiences related to a particular phenomenon to be presented in a direct and comprehensive manner while remaining close to the meanings expressed by participants.^29^ This approach was chosen because the study aimed not to seek the essence of a phenomenon or to generate theory, but rather to describe school personnel's practice-oriented perceptions and experiences related to the return-to-school process. A qualitative descriptive approach was also considered appropriate because the study sought to generate clinically and practically relevant knowledge that could inform future nursing-informed and interdisciplinary planning for school reintegration. In line with this design, the analysis was kept close to participants' accounts and focused on low-inference descriptions of what school personnel observed, needed, and recommended during school re-entry, rather than on developing an interpretive theory or phenomenological explanation. The study was reported in accordance with the Consolidated Criteria for Reporting Qualitative Research (COREQ) checklist.^30^ The COREQ checklist was used to guide the reporting of the research team and reflexivity, study design, participant recruitment, data collection, data analysis, and presentation of findings.

### Setting and participants

The study sample consisted of 12 school personnel who had been in contact with a student returning to school after an interruption of at least 6 months due to childhood cancer and who had played a role in the student's educational and/or psychosocial adjustment. The 6-month interruption criterion was used as an operational inclusion criterion to ensure that participants had experience with a prolonged disruption in schooling and a substantive return-to-school process. It was not intended to define a universal clinical or educational threshold for school re-entry. The participants included eight teachers, two school administrators, one school counselor, and one school nurse. Including participants from different professional roles was intended to capture the multidimensional nature of the return-to-school process from multiple perspectives. The term “contact” referred to active involvement in the student's return-to-school process, such as classroom teaching, academic adjustment, psychosocial counseling, peer preparation, family-school communication, administrative planning, health monitoring, or hospital-school educational support. It did not refer to a single or incidental encounter.

The study was not designed as a stratified comparison by child age group, school level, duration of school interruption, participant role, or institution. Instead, purposive maximum variation sampling was used to capture diverse school-side experiences of return to school. Therefore, differences related to child age, school level, number of prior re-entry experiences, and institutional setting were documented as contextual characteristics rather than used as stratification variables. These contextual details are presented in Table 1 to support interpretation and transferability of the findings.

**Table 1:** Descriptive characteristics of the participants (*N* = 12).

No.	Role	Institution/ setting	Age	Sex	Education	Years in profession	Cancer training	Number of school re-entry cases supported/discussed	Age/school level of students	Duration of school interruption of students	Time since most recent re-entry experience	Role/contact during re-entry process
P1	Teacher	High school	35–44 years	F	PhD	> 10 years	No	2	13 and 15 years; High school level	10 and 22 months	26 months before the interview	Classroom teaching, academic adjustment, peer/classroom observation
P2	Teacher	Primary school	35–44 years	M	Bachelor's degree	> 10 years	No	1	9 years; primary school level	8 months	10 months before the interview	Classroom teaching, academic support, observation of learning loss
P3	Teacher	Primary school	≥ 45 years	F	Bachelor's degree	Between 5 and 10 years	Yes	1	8 years; primary school level	7 months	4 months before the interview	Classroom teaching and follow-up of academic/social adjustment
P4	Teacher	Hospital school affiliated with pediatric oncology clinic	25–34 years	F	Bachelor's degree	Between 5 and 10 years	No	Multiple students receiving oncology treatment	Various age groups; across different school levels	Varied; at least 6 months for the re-entry cases discussed	Ongoing during the study period	Hospital-school educational support during treatment and transition planning
P5	Teacher	Primary school	25–34 years	M	Bachelor's degree	Between 5 and 10 years	No	1	10 years; primary school level	16 months	15 months before the interview	Classroom teaching, peer relationship observation, family communication
P6	Teacher	High school	35–44 years	F	Bachelor's degree	> 10 years	No	1	15 years; high school level	9 months	11 months before the interview	Classroom teaching, emotional support, observation of adolescent adjustment
P7	Teacher	High school	≥ 45 years	F	Bachelor's degree	> 10 years	No	1	18 years; high school level	22 months	8 months before the interview	Classroom teaching, academic adaptation, fatigue/attention observation
P8	Teacher	Hospital school affiliated with pediatric oncology clinic	25–34 years	M	Bachelor's degree	Between 5 and 10 years	No	Multiple students receiving oncology treatment	Various age groups; across different school levels	Varied; at least 6 months for the re-entry cases discussed	Ongoing duringthe study period	Hospital-school educational support and continuity of learning during treatment
P9	School counselor	High school	25–34 years	F	Master's degree	Between 5 and 10 years	No	2	Adolescent; high school level	At least 6 months for both students	Ongoing during the study period	Psychosocial counseling, peer preparation, school adjustment support
P10	School administrator	High school	≥ 45 years	M	Bachelor's degree	> 10 years	No	Multiple students	Adolescent; high school level	Varied; at least 6 months for the re-entry cases discussed	Ongoing during the study period	Administrative planning, family-school communication, school arrangements
P11	School administrator	High school	≥ 45 years	M	Bachelor's degree	> 10 years	No	Multiple students	Adolescent; high school level	Varied; at least 6 months for the re-entry cases discussed	Ongoing during the study period	Administrative coordination, school policy/practice arrangements
P12	School/infirmary nurse	High school	25–34 years	F	Bachelor's degree	< 5 years	Yes	Multiple students	Adolescent; high school level	Varied; at least 6 months for the re-entry cases discussed	Ongoing during the study period	Health monitoring, infection/fatigue-related support, communication with school staff

**Note:** P4 and P8 were hospital-school teachers, not health care professionals, and had experience supporting multiple students during cancer treatment and transition to regular school settings. All regular-school participants worked in different schools, and no overlap was identified among their accounts based on the recruitment records. At least nine separate school re-entry experiences were distinguishable; however, the exact number of unique child-level cases could not be determined because some participants described multiple students and the two hospital-school accounts may have overlapped. For participants with multiple-student experience, the table reflects the cases discussed during the interviews. All discussed re-entry cases involved an interruption of at least 6 months to regular schooling. No direct student identifiers were recorded.

Two participants, P4 and P8, were teachers working in a hospital school affiliated with the pediatric oncology clinic. They were not health care professionals or clinical staff. They were included because they had direct experience in maintaining educational continuity during cancer treatment and observing the transition from hospital-based education to regular school settings. Their perspectives differed from those of regular school teachers because they were involved during the treatment period and could describe educational continuity, academic disconnection, and transition needs before full return to the child's own school. Therefore, their accounts were interpreted as reflecting educational continuity during treatment and transition, rather than as equivalent to full reintegration into the child's regular classroom. In the analysis and reporting, hospital-school support, home-based educational continuity, and full return to the child's regular school were treated as related but distinct aspects of the broader school re-entry process.

Purposive sampling, specifically maximum variation sampling, was used, and snowball sampling was applied when needed to facilitate access to information-rich participants.^31^ Families of children and adolescents who were receiving or had completed cancer treatment and had experienced a cancer-related interruption to schooling were initially contacted through the hospital. Children and their families identified or suggested school personnel involved in the child's educational continuity or school re-entry, and families provided verbal permission solely to facilitate contact. Before sharing any contact information, families asked the suggested school personnel whether they agreed to have their contact details provided to the researchers. Only the contact information of those who agreed was shared. The researchers then contacted these individuals, introduced the study, and obtained written informed consent from those who voluntarily agreed to participate. Children and families were not study participants, and no data were collected directly from them; therefore, formal child assent was not obtained. Interviews focused on school personnel's professional experiences. Only the student's age group and duration of absence from school were recorded as contextual information without direct identifiers. No student names, contact details, medical-record information, or other direct identifiers were collected, and no medical or school records were accessed. In addition, participating school personnel were asked to suggest other eligible individuals with similar experience, and snowball sampling was used when appropriate. Teachers working in the hospital school affiliated with the pediatric oncology clinic were also invited because of their direct experience in supporting educational continuity during treatment and transition to regular school settings. A total of 19 school personnel were approached and screened through three recruitment routes. Through the family-mediated route, six school personnel were approached; all six met the eligibility criteria and participated. Two hospital-school teachers were approached directly, and both were eligible and participated. Through snowball sampling, 11 individuals were approached and screened. Six met the eligibility criteria: four participated, including one school nurse, while two teachers could not be interviewed because of workload and scheduling difficulties. The remaining five individuals reached through the snowball route were school nurses who did not meet the eligibility criterion of having direct experience with the school re-entry process of a child with cancer. Overall, 14 individuals were eligible, 12 participated, two eligible individuals could not participate because interviews could not be scheduled, and five were ineligible. No participant withdrew after providing consent. Recruitment through multiple routes continued until participants from different school roles and institutional settings were included, enabling exploration of the academic, administrative, psychosocial, and health-related dimensions of school re-entry. However, because participation was voluntary, the possibility of volunteer selection bias cannot be completely excluded.

Detailed information on participants' roles, institutional settings, number of school re-entry cases involving children with cancer, age/school level of the child or children, duration of school interruption, time since the most recent re-entry experience, and nature of contact during the re-entry process is presented in Table 1. This information provides contextual detail on the scope of participants’ experience and the diversity of school-based perspectives represented in the study. Participants were from different school-related settings rather than a single school. Because schools may differ in their institutional resources, policies, and procedures for supporting students with health-related needs, institutional setting was documented in Table 1 and considered during interpretation. However, the study was not designed to compare policies or procedures across schools or institutions.

The study was based on school personnel's accounts of school re-entry-related experiences rather than on a child-level case registry. All regular-school participants worked in different schools, and no overlap was identified among their accounts based on the recruitment records. Among participants who described experiences involving one or two clearly distinguishable students, at least nine separate school re-entry experiences were represented across the interviews. In addition, the two hospital-school teachers and some participants in administrative or other school-based roles described experiences involving multiple students. Because the two hospital-school teachers worked in the same hospital school affiliated with the pediatric oncology clinic, their accounts may have included some of the same students. Therefore, the exact total number of unique child-level cases represented across all interviews could not be determined with certainty. Accordingly, the analysis focused on school personnel's perspectives rather than on enumerating individual child cases.

Sample size was determined based on data saturation, as recommended in qualitative research. Data collection and preliminary analysis were conducted concurrently, and recruitment was continued until sufficient thematic depth and repetition were observed across interviews. Saturation was assessed by the two researchers involved in the analysis through regular review of emerging codes, patterns, and thematic categories after each set of interviews. By the later interviews, no substantially new codes were identified and additional interviews primarily elaborated already established categories; on this basis, the research team judged that sufficient analytic saturation had been reached and data collection was concluded. No additional participants were recruited after the research team determined that sufficient analytic saturation had been achieved. Saturation was assessed at the level of the overall thematic structure rather than separately for each professional subgroup. Therefore, the study does not claim role-specific saturation for school counselors or school nurses, each of whom was represented by one participant. During recruitment, six nurses working in or related to school or school infirmary settings were contacted; however, only one met the inclusion criteria and had direct experience with the school re-entry process of a child with cancer. Therefore, the school nurse was included to support maximum variation and to reflect the health-related dimension of school re-entry, but role-specific saturation was not claimed for this subgroup. These roles were included to provide maximum variation and to make visible psychosocial and health-related dimensions of school re-entry, but their limited representation was considered when interpreting the findings and is acknowledged as a limitation. Inclusion criteria were willingness to participate voluntarily, active involvement in the educational process of a student who had been absent from school for at least 6 months due to cancer treatment, and sufficient cognitive and physical ability to participate in an interview. School personnel who had no direct experience with or active role in the student's return-to-school process were excluded.

### Data collection tools

Data were collected using a Descriptive Characteristics Form and a semi-structured individual interview guide developed by the researchers in line with the literature. The Descriptive Characteristics Form included questions on participants' age, sex, educational background, professional experience, role within the school, and prior cancer-related training and school re-entry experience, including the number of children with cancer re-entry experience, the age/school level of the child or children, duration of school interruption, time since the most recent re-entry experience, and the participant's role or contact during the re-entry process.

The semi-structured interview guide was developed by two faculty members with PhD degrees in pediatric nursing who had research experience in pediatric oncology and qualitative research. The guide was based on key literature examining return to school, prolonged school absence, educational continuity, psychosocial adjustment, peer relationships, family-school-healthcare communication, and information needs during school reintegration.^3^, ^4^, ^5^^,^^7^, ^8^, ^9^, ^10^, ^11^^,^^26^, ^27^, ^28^ This literature was considered relevant because it addressed the same phenomenon of school re-entry for children and adolescents undergoing or having completed childhood cancer and focused on barriers, facilitators, support needs, and stakeholder experiences related to the child's return to school. Although not all of these studies focused exclusively on school personnel, they informed the development of questions that could elicit school-side perspectives on academic, psychosocial, health-related, and coordination aspects of re-entry. After the initial draft was prepared, expert opinions were obtained from one faculty member with expertise in qualitative research and three faculty members with PhD degrees in pediatric nursing who had experience in pediatric oncology. These experts reviewed the guide for clarity, relevance, comprehensiveness, and appropriateness for eliciting school personnel's experiences of return to school. Based on their feedback, the wording and flow of the questions were refined.

The interview guide was organized around school personnel's concrete experiences of school re-entry, including academic adjustment, peer relationships, family-school communication, health-related concerns, and information needs. The main interview areas included the participant's role in the re-entry process, observed academic and social changes in the student, challenges encountered by the student, family, peers, and school personnel, school-based accommodations or support practices, communication with the family and health care team, perceived information gaps, and suggestions for improving the return-to-school process. The main domains of the semi-structured interview guide and sample questions are presented in Supplementary Table S1. It also included prompts about communication with families and health care professionals; however, it did not include a separate question specifically naming pediatric oncology nurses. For this reason, the study does not present nursing-related implications as direct participant requests, but rather interprets them cautiously as potential implications derived from school personnel's broader accounts of information, communication, and coordination needs.

### Data collection procedure

Following ethics committee approval, school personnel who met the sampling criteria were contacted and informed about the study. After written informed consent was obtained from those who agreed to participate, the Descriptive Characteristics Form was completed and an appropriate time was scheduled for the individual interviews. All recruitment and data collection procedures were conducted after ethics committee approval had been obtained. Data collection was carried out between April 2025 and January 2026. Data were collected online via Zoom using the semi-structured interview guide developed in line with the literature. Online interviews were preferred because participants were located in different school or hospital-school settings and because this format allowed interviews to be scheduled at times convenient for school personnel without disrupting school routines. The use of Zoom also facilitated participation by reducing travel and time burden. To support rapport and data quality in the online environment, the interviews were conducted at participant-preferred times, the purpose of the study was explained clearly before the interview, open-ended questions and flexible probes were used, and participants were encouraged to describe concrete examples from their own experiences.

Two researchers attended all interviews; the first researcher conducted the interviews, while the second observed the process and took notes. At the beginning of each interview, the researchers explained their roles: one researcher would lead the interview, while the other would remain mostly silent, observe the flow of the interview, and take field notes. This explanation was provided to minimize participant discomfort or the feeling of being evaluated. Participants were also reminded that there were no right or wrong answers and that they could decline to answer any question or withdraw at any time. Interviews were conducted at times convenient for the participants, in quiet and uninterrupted settings, and each interview lasted approximately 30–45 minutes. No non-participants were present during the interviews. Completion of the Descriptive Characteristics Form took approximately 5 minutes and was conducted before the interview; therefore, the reported 30–45-min duration refers to the semi-structured interview itself. The interview duration was sufficient because the interviews were focused on participants’ concrete experiences of school re-entry, while allowing additional probes when clarification or examples were needed. Each participant was interviewed once, and no repeat interviews were conducted.

With participants’ permission, audio recordings were made, and the recordings were transcribed verbatim after each interview. The notes taken during and immediately after each interview were reviewed together with the participant at the end of the same interview session, allowing participants to clarify, elaborate on, or correct the recorded information. This process served as end-of-interview meaning validation, ensuring that the researchers' understanding accurately reflected the participants' intended meanings. Full interview transcripts were not returned to participants for review. Emotional tone, hesitation, emphasis, and visible non-verbal cues observable through Zoom, such as facial expression and pauses, were also noted when relevant. Because this was a qualitative meaning-validation process rather than a quantitative reliability assessment, no quantitative consistency score was calculated; however, the same end-of-interview review procedure was applied across all interviews.

The two researchers who attended the interviews were female academic researchers with PhD degrees in pediatric nursing and had experience in pediatric oncology and qualitative research. Their professional background provided sensitivity to issues related to childhood cancer and family-centered care; to manage this potential influence, collaborative analytic discussions were conducted throughout data collection and analysis, and field notes were used. There was no hierarchical or direct work relationship between the researchers and the participants. The involvement of two pediatric nursing researchers during the interviews also supported attention to issues relevant to the cancer care continuum, psychosocial adjustment, family-centered care, and potentially nursing-relevant aspects of school-side information and coordination needs.

### Data analysis

Data were analyzed using a team-based inductive thematic analysis informed by Braun and Clarke's six-phase framework.^32^ Two researchers independently coded the transcripts, discussed and refined the coding through consensus, and collaboratively developed the themes. The analysis process involved repeated reading of the transcripts to achieve familiarity with the data, generating initial codes from meaningful data segments, grouping similar codes into candidate themes, reviewing the themes against the data set, naming the themes and subthemes, and finally reporting the findings within an analytic narrative. In the first phase, both researchers read the transcripts several times and noted preliminary impressions. In the second phase, meaningful statements related to school re-entry experiences, observed student needs, family-school communication, peer relationships, health-related concerns, and support practices were coded inductively. In the third and fourth phases, related codes were grouped into candidate themes and then reviewed against the full data set to ensure that each theme was internally coherent and clearly distinguishable from the others. In the fifth phase, the final analytic domains, themes, and subthemes were named to reflect school personnel's perspectives as closely as possible. In the final phase, the findings were written with illustrative quotations and an analytic narrative linking the themes to the study aim.

The analysis was conducted using an inductive and data-near descriptive approach aimed at summarizing participants' reported experiences, observations, concerns, and suggestions in their own terms as closely as possible. Consistent with the qualitative descriptive design, coding was primarily low-inference and remained close to participants' descriptions of observed challenges, support needs, communication gaps, and school-based practices. The final structure was organized as analytic domains, themes, and subthemes rather than as a theory-building framework. The analytic domains were used to group related themes at a broader descriptive level, while the themes and subthemes represented more specific patterns in participants’ accounts.

Coding and theme development were undertaken independently by two researchers, after which the codes and themes were discussed until consensus was reached. This process was used not to achieve coder agreement in a quantitative sense, but rather to deepen interpretations of the data, consider alternative meanings, and analytically refine the thematic structure. Throughout the analysis, continuous movement between the data and the emerging themes was maintained, and theme boundaries and labels were repeatedly reviewed until the final thematic structure was established. Particular attention was paid to distinguishing conceptual boundaries between themes so that individual, relational, and institutional aspects of school reintegration were analytically separable and clearly interpretable. Special attention was given to presenting the findings as school personnel's perspectives rather than as a generalized description of the school re-entry process itself. The researchers also reviewed whether each theme was sufficiently supported by participant quotations and whether claims in the Discussion were grounded in the Results. During analysis, the researchers also considered how the issues raised by school personnel reflected support needs that could be relevant to future nursing-informed interdisciplinary support, while keeping the coding process grounded in participants' own accounts.

Differences across institutional settings and participant roles were considered during analysis by comparing whether similar or different concerns were expressed by regular school teachers, hospital-school teachers, administrators, the school counselor, and the school nurse. These comparisons were used to refine theme boundaries and to avoid treating all school personnel as a homogeneous group. However, these comparisons were used for contextual interpretation and analytic refinement rather than for formal subgroup comparison, because the study was not designed to compare participant roles or institutional settings. No qualitative data analysis software was used; coding and theme development were conducted manually through repeated reading, independent coding, comparison, discussion, and analytic notes.

### Rigor

To ensure rigor, the study was guided by Lincoln and Guba's criteria of credibility, transferability, dependability, and confirmability.^33^ Credibility was enhanced through detailed transcription of the interviews and repeated return to the data by the researchers to review the coding and thematic structure. Credibility was also supported by participant meaning validation at the end of each interview, use of probes to clarify unclear statements, and comparison of emerging codes across interviews during concurrent data collection and analysis. The review of the coding and theme structure by two researchers also helped enhance credibility by allowing alternative interpretations to be discussed before the final thematic structure was established.

Transferability was supported by providing a detailed description of participant characteristics and the research context. Table 1 was expanded to provide more detailed contextual information about participants’ roles, institutional settings, experience with children with cancer returning to school, age/school level of the child or children, duration of school interruption, time since the most recent re-entry experience, and the nature of contact during the re-entry process. These details were provided to allow readers to judge whether the findings may be relevant to other school or pediatric oncology contexts, rather than to claim statistical generalizability.

Dependability was strengthened by having two researchers conduct the analysis independently and by documenting analytical decisions throughout the process. Analytical decisions, changes in coding, theme merging or separation, and disagreements between researchers were documented through analytic notes. The use of the same semi-structured interview guide, consistent end-of-interview meaning validation, concurrent data collection and analysis, and documentation of coding decisions also supported dependability.

Confirmability was enhanced by obtaining input from an expert who was not directly involved in the study and by sharing summaries of participants' accounts at the end of the interviews for meaning validation.^33^^,^^34^ The coding and theme structure were reviewed by an independent faculty member with expertise in qualitative research who was not directly involved in data collection or primary analysis. This external review focused on the clarity, coherence, and grounding of the coding structure and theme organization. Confirmability was further supported through reflexive discussions, field notes, and an audit trail documenting how the final themes were developed from participants’ accounts.

In addition, notes taken during and after the interviews were used to support contextual interpretation, and records related to theme development were retained to enhance the traceability of analytical decisions. Transferability was addressed not as a claim of generalizability to other contexts, but by presenting the study context and participant characteristics in sufficient detail to enable readers to make judgments about relevance to similar settings. To enhance transparency regarding sample adequacy, decisions related to saturation were documented through ongoing analytic discussions between the two researchers during the data collection and analysis process. Differences across participant roles and institutional settings were considered during analysis by comparing whether similar issues appeared across different school contexts or whether certain concerns were linked to a specific role or setting. This process was used to refine the interpretation of the findings, but not to produce role-specific or institution-specific comparisons.

The researchers also engaged in reflexive discussions to identify how their pediatric oncology nursing background might shape interpretation. Although formal phenomenological bracketing was not the aim of this qualitative descriptive study, reflexive bracketing strategies were used to make researchers' assumptions visible and to reduce the risk of imposing a nursing-centered interpretation on school personnel's accounts. For example, when interpreting participants' accounts of information gaps and coordination needs, the researchers discussed whether these accounts represented direct expectations of nurses or broader school-side support needs. As a result, nursing implications were framed cautiously as school-side needs that could indicate potential areas in which pediatric oncology nurses may be involved in future interdisciplinary communication and coordination, rather than as direct demands expressed by participants. When participants described uncertainty about health-related information, the researchers avoided coding these accounts as direct expectations from pediatric oncology nurses unless such expectations were explicitly stated; instead, these accounts were coded descriptively as school personnel's information and coordination needs.

## Results

### Participant characteristics

A total of 12 participants representing different professional roles and school levels were included in the study. The participants consisted of teachers, school administrators, a school counselor, and a school nurse, and they varied in age, sex, educational background, professional experience, and cancer-related experience. The descriptive characteristics of the participants, including their professional roles, institutional settings, school re-entry-related experience, age/school level of the children/adolescents, duration of school interruption, time since the most recent re-entry experience, and nature of contact during the re-entry process, are presented in Table 1.

### Analytic domains and themes

As a result of the interview analysis, the findings related to school re-entry were organized under three analytic domains and eight themes. To clarify the school-side perspective, the findings are presented not as a generalized description of school reintegration, but as school personnel's observations, concerns, perceived support needs, and recommendations regarding the school re-entry process. Overall, the findings showed that return to school was experienced not merely as re-engagement with academic life, but as a multidimensional process involving academic and health-related adjustment, social and emotional reintegration, and school-side support and coordination needs. The three analytic domains were organized to distinguish: (1) school personnel's observations of academic and health-related adjustment, (2) school personnel's perceptions of social and emotional reintegration, and (3) school-side support needs and coordination gaps. The analytic domains, themes, and subthemes are presented in Table 2.

**Table 2:** Analytic domains, themes, and subthemes of school re-entry during or after childhood cancer treatment.

Analytic domain	Theme	Subthemes
School personnel's observations of academic and health-related adjustment	The losses of school absence	● Disruption of academic continuity and learning loss ● Feelings of being left behind, performance pressure, and diminished self-confidence ● Social withdrawal and disruption of daily life routines
	Health-related vulnerability and individualized adjustment	● Infection risk and ongoing physical sensitivity ● Fatigue, limited endurance, and environmental sensitivity ● Need for individualized adjustments in academic and physical processes
	Home-based education as a bridge	● Home-based education serving as a bridge that prevents complete disconnection from school ● Relationships maintained with familiar teachers providing trust and continuity ● Preservation of student identity and school belonging
School personnel's perceptions of social and emotional reintegration	Restoring belonging and normalcy	● Emotional safety taking precedence over academic expectations ● School gaining meaning as a space for returning to normalcy and everyday life ● Gradual readjustment to school life
	Peer acceptance and visible difference	● Fear of exclusion, stigmatization, and being mocked ● The impact of visible bodily changes on self-perception and social confidence ● Preparing the classroom and fostering peer awareness
	Shared emotional burden and support needs	● The family's psychological, caregiving, and logistical burden ● School personnel's search for support in the context of limited information ● The emotional labor of teachers and counseling staff
School-side support needs and coordination gaps	Support shaped by individual effort	● Support being provided largely through individual dedication and voluntariness ● The facilitating or constraining role of school culture ● Patterns of support varying across institutions
	Shared responsibility and coordinated planning	● The need for school-family-hospital collaboration ● The need for joint planning among school administration, teachers, counseling services, and the school/infirmary nurse ● A child-specific structured readjustment plan and follow-up

#### Analytic domain 1. School personnel's observations of academic and health-related adjustment

This analytic domain includes the themes that participants described as most directly related to the child's academic continuity, school participation, and health-related adaptation. Participants' accounts showed that this process was shaped around three main themes: the multidimensional losses associated with being away from school, health-related vulnerability and the need for individualized adjustment, and home-based education as a bridge. When considered together, these themes indicate that school personnel viewed return to school as requiring practical, individualized, and health-sensitive arrangements rather than a simple resumption of classroom attendance. Home-based education was included in this analytic domain because participants primarily described it as a structural and educational bridge that helped maintain school connection, reduce academic disconnection, and prepare the child for later classroom participation. In this domain, home-based education and hospital-school support were interpreted as forms of educational continuity during treatment and transition, rather than as equivalent to full reintegration into the child's regular classroom.

##### Theme 1. The losses of school absence

Participants emphasized that time away from school created interrelated academic, emotional, and social losses in the child's life. This theme was shaped around three main dimensions: disruption of academic continuity, loss of self-confidence associated with feeling left behind, and social withdrawal. Long-term absence disrupted the child's academic rhythm and continuity of learning, creating a strong perception of falling behind peers. This sense of disconnection extended beyond lessons and was described as affecting the child's confidence, motivation, and sense of competence as a student. Academic disconnection was also associated with increased performance pressure, anxiety about catching up, and reduced self-confidence. At the same time, being away from school and the peer environment appeared to be associated with weakened friendships, social withdrawal, and disruption in daily life routines. Taken together, these accounts showed that absence from school was experienced not only as missed instruction, but as a broader loss of routine, confidence, and social participation. From the perspective of school personnel, absence from school was therefore viewed as a multidimensional disruption that affected academic continuity, student identity, peer connectedness, and participation in everyday school routines.

Participant statements supporting this theme indicated that children often experienced being away from school through feelings of “falling behind” and “not being able to catch up.” One hospital-school teacher described how prolonged absence from regular school could create a marked decline in the learning process:

*“Even without home-based education, when children are away from school for a long time, we very clearly see that they fall behind academically.”* (P8, Hospital-school teacher)

Similarly, a school administrator emphasized that academic disconnection created not only a learning gap, but also a strong sense of inadequacy and performance pressure in the child:

*“What they experience most is the feeling of being left behind. This becomes more visible especially for successful and ambitious students. They may feel things like, ‘My friends are ahead of me,’ ‘I won’t be able to catch up,’ or ‘I no longer have my old level of performance.’”* (P10, School administrator)

These accounts suggest that school absence was understood by participants as a multidimensional disruption that affected not only academic progress but also the child's confidence, peer connectedness, and continuity of everyday school life.

##### Theme 2. Health-related vulnerability and individualized adjustment

Participants emphasized that the return-to-school process cannot be considered independently of ongoing health-related vulnerabilities. This theme was shaped around three main dimensions: infection risk and ongoing physical sensitivity, environmental sensitivity associated with fatigue and limited endurance, and the need for individualized adjustments in academic and physical processes. Children's vulnerabilities related to their immune system, fear of contracting infections, exposure to crowded environments, physical fatigue, and rapid exhaustion directly affected school participation. Participants described these vulnerabilities as shaping not only whether the child could attend school, but also how the child could participate in classroom routines, physical activities, and the overall daily flow of school life. These vulnerabilities required not only monitoring of health status, but also the reorganization of classroom location, break arrangements, participation in physical activities, hygiene conditions, lesson load, and the daily flow of school according to the child's individual needs. In this respect, return to school was portrayed as an individualized process of adjustment that needed to be responsive to the child's health status, capacity, and treatment-related sensitivities. Taken together, the accounts suggested that school participation was closely tied to the recognition of health-related limits and the flexibility of the school environment in responding to them. School personnel therefore emphasized the need for concrete, school-useable information about what the child could safely do, what should be monitored, and how classroom routines could be adapted.

Participant statements supporting this theme showed that health safety and physical tolerance were among the main determinants of the return-to-school process. A school nurse highlighted infection risk and ongoing physical sensitivity as key concerns in everyday school life:

*“I would place infection risk first. Because even if treatment has ended, many students' immune systems can remain very sensitive for a while. Even the simplest upper respiratory tract infection can become significant for them.”* (P12, School nurse)

Participants' accounts also clearly revealed the need for individualized accommodations. One teacher explained that school-based arrangements should be made flexibly according to the child's health condition:

*“They get tired very quickly. They forget very quickly too, even though C. is normally a very successful child. Under normal circumstances, if something like this had not happened, this could have been a child who might even have been the top student in the school. Yes, they are very hardworking and very ambitious, but they get tired very quickly. And they still have difficulties with maintaining focus in the classroom.”* (P7, Teacher)

These accounts indicate that return to school was not experienced as a uniform process, but as one requiring individualized adjustments based on the child's physical tolerance, ongoing health risks, and changing capacity to participate in school life.

##### Theme 3. Home-based education as a bridge

Participants emphasized that home-based education and hospital-school educational support functioned as protective transition mechanisms that enabled the child to maintain a connection with school during the return-to-school process. Within this theme, issues such as home-based education or hospital-school support serving as a bridge that prevents complete disconnection from school, the relationship maintained with familiar teachers providing trust and continuity, and the preservation of student identity and school belonging came to the forefront. Teachers stated that these forms of educational continuity not only prevented the child from becoming completely disconnected from lessons during long periods outside school, but also contributed to maintaining the child's relationship with school at symbolic and emotional levels. In particular, continued contact with their own teachers or hospital-school teachers prevented the child from feeling forgotten or excluded and helped preserve the sense of belonging to a school community. At the same time, home-based education and hospital-school support were experienced not only as the delivery of academic content, but also as meaningful support mechanisms that allowed the child to maintain the feeling of “I am still a student.” In this respect, these forms of educational continuity served as protective bridges before or alongside later return to the child's regular school, rather than representing full regular school reintegration in themselves. Participants' accounts suggested that continuity of contact during treatment reduced the risk of complete relational rupture and made later school return more manageable. Although this theme includes relational elements such as trust and student identity, participants primarily framed home-based education and hospital-school support as school-based continuity mechanisms that reduced academic disconnection and supported later classroom re-entry in the child's regular school setting.

Participant statements supporting this theme indicated that home-based education and hospital-school support were seen as processes that prevented the child from becoming completely disconnected from school and facilitated later readjustment. A hospital-school teacher described how teachers' home visits and continuity of contact helped keep the child's relationship with school alive:

*“They never felt alone. It was very important that their teachers visited their home and that the school did not break its bond with them. I think this was one of the most important things that made their return to school easier.”* (P8, Hospital-school teacher)

Similarly, one teacher emphasized that home-based education is important not only for academic support, but also for preserving student identity:

*“When home-based education is provided, the child does not become completely disconnected from school. At the very least, they continue to feel that they are still a student of that class and that school. This also makes adjustment easier when they return.”* (P7, Teacher)

These accounts suggest that home-based education functioned not only as academic continuity, but also as a relational bridge that preserved trust, student identity, and connection to the school community.

#### Analytic domain 2. School personnel's perceptions of social and emotional reintegration

This analytic domain includes the themes that participants associated with the child's emotional safety, sense of belonging, peer relationships, visible difference, and the emotional burden shared by the child, family, and school personnel. Participants' accounts showed that this process was shaped around three main themes: restoring belonging and normalcy, peer acceptance and visible difference, and shared emotional burden and support needs. Together, these themes indicate that school personnel viewed social and emotional reintegration as a school-side responsibility involving emotional safety, classroom climate, peer preparation, and recognition of the emotional needs of both families and school staff. Participants expressed that successful school return depended not only on the child's willingness to come back, but also on whether the surrounding relational environment was prepared to receive and support that return.

##### Theme 4. Restoring belonging and normalcy

Participants described return to school as an emotional and developmental process of readjustment in which the child begins to feel like part of the school again. This theme was shaped around three main subdimensions: emotional safety taking precedence over academic expectations, school gaining meaning as a space for returning to normalcy and everyday life, and gradual readjustment to school life. According to participants, the first priority in the return-to-school process was not the child's academic performance, but whether the child felt emotionally safe, accepted, and once again experienced a sense of belonging in the school environment. In this context, school was understood not merely as an institution where lessons continue, but as a symbolic space in which the child reconnects with daily life, social relationships, and a sense of normalcy. In addition, return to school was often experienced not as an abrupt event, but as a gradual and supported process of re-engagement; this gradual structure appeared to facilitate the child's psychological comfort as well as the reconstruction of school belonging. The gradual return referred to here includes maintaining contact with the school during the hospital process, online education, individualized education, and rejoining the classroom. In this respect, belonging points to a multidimensional process of reconstruction that includes reconnecting with student identity and the bond with school. Participants stated that feeling emotionally secure and gradually reconnecting with everyday school life were seen as prerequisites for later academic participation. From the school personnel's perspective, emotional safety and gradual reconnection were viewed as necessary conditions for the child's later participation in academic and social school life rather than as secondary concerns. In this sense, return to school was framed less as an immediate academic recovery and more as a supported re-entry into ordinary life.

Participant statements supporting this theme clearly showed that emotional safety takes precedence over academic expectations. One teacher expressed that, in the initial stage, the child's psychological well-being was prioritized over academic achievement as follows:

*“At school, they need to be welcomed with a smiling face. In the early period, what matters is helping them adapt by talking with them much more than focusing on lessons. Later, we can try to support them in becoming oriented to their lessons. Our main aim is to keep their psychology in a good place. Before trying to help them hold on to life and adapt to lessons, we make efforts directed toward that first.”* (P1, Teacher)

Similarly, a school administrator defined return to school not merely as continuing education, but as a broader process of readjustment:

*“As school administrators, we do not view these children as ‘students who have returned to school,’ but as ‘students trying to readjust to life.’ First, we need to think about their safety, then their psychological comfort, and only then their academic adjustment.”* (P10, School administrator)

These accounts show that participants framed school re-entry as a process in which emotional safety, acceptance, and ordinary school routines needed to be re-established before academic expectations could become central.

##### Theme 5. Peer acceptance and visible difference

Participants emphasized that social adjustment during the return-to-school process was experienced as a particularly fragile area, especially in the context of peer relationships. This theme was shaped around three main dimensions: fear of exclusion, stigmatization, and being mocked; the impact of visible bodily changes on self-perception and social confidence; and the preparation of the classroom and development of peer awareness. According to teachers' statements, when children return to school, they often have to cope with the curious looks, questions, and possible reactions of their friends. In particular, differences such as hair loss, weight change, mask use, or changes in general physical appearance were found to make children feel conspicuous, vulnerable, and open to evaluation. This could increase social withdrawal, avoidance of visibility, and reluctance to rejoin the peer environment in some children. Participants also stated that social adjustment does not develop spontaneously; rather, preparing classmates in advance, increasing peer awareness, and creating an inclusive classroom atmosphere are important for reducing this fragility. In this respect, social adjustment emerged as a relational process shaped not only by the child's individual effort, but also by the attitudes of peers and the school. School personnel therefore viewed peer preparation and classroom climate as actionable areas of school-side support. Rather than being a simple matter of “fitting back in,” peer reintegration was described as a fragile process shaped by how visible difference was interpreted and responded to within the classroom.

Participant statements supporting this theme described children's experiences with peers' attention and exclusionary attitudes following their return to school. One teacher described how visible differences became a source of anxiety for the child:

*“There were times when they did not want to come because they thought they would be mocked. Especially during the period when they had no hair, they became much more withdrawn. They thought a lot about what their friends would say.”* (P5, Teacher)

Similarly, a school counselor emphasized the determining role of peer acceptance in the social adjustment process:

*“One of the most difficult things for the child is how their friends will look at them. If the class accepts them as they are, adjustment becomes much easier; but if they feel they will not be accepted, they can withdraw very quickly.”* (P9, School counselor)

These accounts indicate that peer acceptance was not a peripheral issue, but a central condition shaping whether the child could re-enter the social life of school with confidence.

##### Theme 6. Shared emotional burden and support needs

Participants emphasized that the return-to-school process involves an emotional burden shared by the child, family, teachers, and other supportive school personnel. This theme was shaped around three main dimensions: the psychological, caregiving, and logistical burden on the family; school personnel's search for support in the context of limited information and the emotional labor of teachers and guidance staff. Participants perceived that families carried intense anxiety, uncertainty, and caregiving responsibility while trying to support the child's re-engagement in school after treatment. Participants did not present family burden as a separate caregiver-focused finding; rather, they described how the family's emotional and practical burden became visible within school communication and affected the support needs encountered by school personnel. At the same time, teachers and school personnel may sometimes feel inadequate regarding how to approach the child, how to provide support in different situations, and how to manage health-related sensitivities. In this process, school personnel were found not only to provide academic support, but also to witness the emotional burden of the child and family, to share part of that burden, and at times to be psychologically affected by it. Participants' accounts suggested that return to school created support needs not only for the child, but also for the family and school personnel accompanying the process. Thus, this theme reflects school personnel's perception of emotional burden as something encountered and managed within school relationships, rather than a generalized account of family caregiving. In this respect, return to school emerged as a relational experience that makes visible the support needs of all stakeholders accompanying the process.

Participant statements supporting this theme showed that families often carry a heavy but largely invisible burden during the return-to-school process. One participant described how mothers focused entirely on the process and pushed their own well-being into the background:

*“Mothers devote themselves completely to the child. They often put their own needs, their own health, and even what they are going through psychologically in second place. In fact, they also need very serious support.”* (P5, Teacher)

Similarly, one teacher emphasized that this process also leaves an emotional impact on school personnel:

*“Sometimes, when the student talks about what they have been through, we are deeply affected as well. Listening to them, knowing what they have experienced, and seeing that they are trying to readjust inevitably wears you down emotionally too.”* (P6, Teacher)

These accounts indicate that school reintegration was experienced not only as the child's transition, but also as a shared emotional process that generated information, guidance, and support needs across the family and school environment.

#### Analytic domain 3. School-side support needs and coordination gaps

This analytic domain includes the themes that participants described in relation to how schools organize, sustain, and coordinate support for students' re-entry. School reintegration was shaped not only by the child's needs, but also by the school's capacity to mobilize support, define responsibilities, and communicate with families and health care teams. Participants' accounts revealed that this process was organized around two main themes: support shaped by individual effort and shared responsibility and coordinated planning. Taken together, these themes indicate that return to school is often carried out through individual dedication and goodwill; however, more structured, visible, and sustained coordination is needed among school administration, teachers, counseling services, school health services, the family, and the health care team for effective and sustainable reintegration. From the perspective of school personnel, successful school reintegration depended not only on the presence of supportive individuals, but also on whether the school could transform individual sensitivity into a consistent, coordinated, and shared support process.

##### Theme 7. Support shaped by individual effort

Participants emphasized that the support provided during the return-to-school process was often shaped less by a formal and standardized system than by individual dedication, voluntariness, and relational labor within the school. This theme centered around issues such as support being provided largely through personal commitment and willingness, the facilitating or constraining role of school culture, and patterns of support that varied across institutions. The sensitivity of teachers, administrators, and other school personnel directly influenced the quality of the process; while in some schools the child and family were actively embraced, in others the same level of support could not be provided. This shows that institutional support is not always equal or predictable, and is often determined by school culture, administrative approach, and the extra efforts of individuals. Rather than being embedded in a clearly defined system, support was frequently described as depending on who happened to take initiative within the school. Thus, institutional sensitivity becomes visible not through standard procedures, but through the relationships established within the school community, the ways responsibility is assumed, and the human sensitivity shown toward the child's needs.

Participant statements supporting this theme showed that support in the return-to-school process was often carried out through personal ownership and voluntariness. A hospital-school teacher described how teachers made efforts that went beyond their formal job descriptions in order not to leave the child unsupported:

*“Teachers visiting the child at home, giving one-to-one attention, and trying not to let the bond break were entirely about taking ownership. This is also somewhat related to the approach of the school personnel; not everyone behaves this way.”* (P8, Hospital-school teacher)

Similarly, a school principal emphasized that support mechanisms may vary from one institution to another and that this can create inequality for children:

*“Supporting these children should not be a matter of luck. If the school principal is sensitive and the teacher is attentive, the process goes better; but when it is left entirely to individuals, not every student receives the same support.”* (P11, School administrator)

These accounts indicate that school reintegration support was often sustained through goodwill and personal commitment rather than through standardized institutional pathways, creating uneven experiences across schools.

##### Theme 8. Shared responsibility and coordinated planning

Participants highlighted collaboration and coordination among multiple stakeholders as important elements in supporting children's return to school and subsequent readjustment following childhood cancer. This theme was shaped around three main dimensions: the need for school-family-hospital collaboration, the need for joint planning among administration, teachers, counseling services, and the school nurse, and the need for a child-specific structured readjustment plan and follow-up. According to participants' accounts, the child's health status, academic capacity, psychosocial needs, and mode of participation in daily school life may be more effectively supported through the sharing of information and responsibility among different stakeholders. In this context, maintaining open communication between the family and school, translating information from the health care team into forms appropriate for the school setting, and developing a shared approach within the school among administration, teachers, counseling services, and the school nurse were seen as important school-side coordination needs. Participants' accounts indicated that schools needed families to share up-to-date information about the child's current condition, emotional readiness, attendance capacity, and changing needs, while participants perceived a need for hospitals and health care teams to provide clear, practical, and school-useable guidance about health-related precautions, activity limitations, fatigue, infection risk, and situations requiring health care contact. Participants also stated that readjustment is not a one-time decision, but a dynamic process that must be monitored according to the child's changing needs and revised when necessary. In this respect, return to school emerged as an area of shared responsibility that requires structured, shared, and continuous coordination beyond individual goodwill. Participants positioned coordination not as an optional addition to school return, but as a core school-side need for making school participation safe, responsive, and sustainable.

Participant statements supporting this theme showed that the most effective approach in the return-to-school process is collaboration and joint planning among stakeholders. A school administrator stated that readjustment can only proceed in a healthy manner when all parties act together:

*“This is a process that the family, school, and hospital need to carry out together. The effort of the teacher alone or the family alone is not enough. Everyone needs to know what they are supposed to do and act together.”* (P10, School administrator)

Similarly, a school nurse emphasized the importance of coordination within the school and ongoing follow-up for a school life that is sensitive to the child's needs:

*“The child’s situation is not something to be discussed once and then left alone. Sometimes things go well, and sometimes it becomes a more sensitive period. That is why the teacher, counseling service, family, and we need to stay in communication. If the process is followed up, the child also feels safer.”* (P12, School nurse)

These accounts suggest that coordinated planning, ongoing communication, and child-specific follow-up were viewed as essential to sustaining the child's adjustment over time. They also show that school personnel did not view hospital and family support as general encouragement alone, but as concrete, timely, and school-oriented information sharing that could help them respond to the child's academic, psychosocial, and health-related needs in everyday school life.

## Discussion

### Main findings

This study showed that, from the perspective of school personnel, return to school is perceived not merely as the resumption of academic attendance, but as a multidimensional process of readjustment intertwined with the student's developmental needs, psychosocial vulnerabilities, peer relationships, and institutional support structures. This finding is consistent with recent literature describing return to school as a complex process encompassing educational, social, and emotional dimensions simultaneously.^3^^,^^35^ The findings further suggest that being away from school affects not only students' ability to keep up with lessons, but also their sense of belonging, their opportunity for synchronous developmental experiences with peers, and their position within the classroom. From this perspective, return to school may be understood less as a simple “resumption of attendance” and more as a process of reconstructing the child's place in school life. The findings of this study may be relevant to supportive care during treatment and long-term follow-up by highlighting school-related challenges that can continue beyond the completion of medical treatment. Although participants did not explicitly discuss the role of pediatric oncology nurses, the challenges they described suggest potential areas for strengthened collaboration between health care and educational professionals. Therefore, the potential nursing-related implications presented in this study should be interpreted as inferred implications derived from school personnel's accounts of information, communication, and coordination needs, and considered alongside the existing literature, rather than as direct expectations expressed by nurses, children, or families. Given that the participants' experiences involved children and adolescents from different school levels, the findings should also be interpreted with attention to developmental differences. School re-entry needs may differ between younger children, who may require more concrete support for routine, safety, and peer understanding, and adolescents, for whom body image, academic performance, autonomy, and peer acceptance may become more prominent. Therefore, the findings support individualized and developmentally sensitive planning rather than uniform recommendations for all age groups.

The main contribution of this study is not only that it confirms the complexity of school re-entry, but that it makes visible how this complexity is perceived and managed from the school side. While previous studies have mostly emphasized the experiences of children and families, the present findings show that school personnel encounter school re-entry as a set of interrelated practical, psychosocial, health-related, and institutional challenges. In particular, the study highlights that school personnel need not only general awareness of childhood cancer, but also clear role definitions, school-useable health information, peer preparation strategies, and sustained communication pathways with families and health care teams. This school-side perspective extends existing knowledge by identifying where continuity between cancer care and everyday school life may weaken.

The first analytic domain, school personnel's observations of academic and health-related adjustment, suggests that students returning to school may not be able to re-enter a standard educational process under the same conditions as before. School personnel's emphasis on students' health-related vulnerabilities, learning losses associated with absenteeism, home-based education as a bridge, and the need for individualized adjustment is consistent with studies reporting that educational support is often delivered in heterogeneous, fragmented, and insufficiently systematic ways.^4^^,^^5^^,^^10^ This finding suggests that return to school should be understood not as a one-size-fits-all practice, but as an individualized transition process that must be planned in light of the student's treatment history, current functioning, and developmental characteristics. In particular, considering that fatigue, attention problems, cognitive effects, or physical limitations may invisibly affect school participation, balancing academic expectations with health-related needs becomes especially important. From a nursing-informed interdisciplinary perspective, these school-side accounts suggest potential areas in which pediatric oncology nurses could be involved in future return-to-school models, particularly by helping translate clinically relevant information into practical school-oriented considerations. These areas may include the child's current functional status, treatment-related sensitivities such as infection risk, fatigue, limited physical endurance, medication-related needs, concentration difficulties, visible bodily changes, emotional vulnerability, and possible accommodations related to classroom participation, activity tolerance, infection prevention, and daily school routines.

A notable aspect of the findings is that time away from school was perceived by school personnel not only as an academic deficit, but also as a multidimensional experience of loss. The student's separation from classroom routines, disconnection from friendships, and estrangement from the everyday flow of school transform return to school from a matter of simply closing curricular gaps into a broader process of re-entry. This interpretation is consistent with recent studies showing that academic catch-up alone is insufficient in school reintegration, and that social placement, coping with visible differences, and the need for renewed acceptance are also decisive.^10^^,^^11^ Therefore, evaluating the return process solely in terms of achievement and attendance indicators may be inadequate; students' sense of belonging at school, self-confidence, and social adjustment should also be considered core components of the transition. In practice, this finding suggests that school-related follow-up may need to move beyond whether the child has physically returned to school and also consider whether the child feels connected, confident, and able to participate meaningfully in school life. These school-side observations could inform future interdisciplinary follow-up models in which pediatric oncology nurses may be involved in helping families recognize school-related concerns and communicate the child's academic, emotional, and social participation needs.

The second analytic domain, school personnel's perceptions of social and emotional reintegration, showed that peer acceptance, visible difference, and shared emotional burden are central to the return-to-school process. School personnel's efforts to adopt a careful and protective stance in response to changes in students' physical appearance, fragile health conditions, or peers' curiosity highlight how fragile social adjustment may be. This finding is supported by qualitative and synthesis studies showing that children and adolescents returning to school may face risks of visible difference, misunderstanding, exclusion, and stigmatization.^35^, ^36^, ^37^, ^38^ For this reason, it does not seem sufficient to leave social reintegration solely to the student's individual coping skills. Teachers' role in shaping the classroom climate, informing peers, and fostering an inclusive school attitude may be decisive for the success of this process. These findings also suggest potential nursing-relevant implications, but these implications should be understood as derived from school personnel's accounts rather than as direct participant requests for nursing action. Although participants did not directly describe expectations from nurses, their accounts highlight school-side needs for anticipatory information, preparation for visible or invisible treatment effects, and communication that may reduce uncertainty, stigma, and social withdrawal. These needs could indicate potential areas in which pediatric oncology nurses may be involved within future interdisciplinary return-to-school models, particularly by contributing to family-school communication and school-oriented explanations of treatment-related changes when appropriate.

Another important finding of this study is that the return-to-school process creates not only a burden for children and families, but also an emotional and professional burden for school personnel. School personnel's efforts to protect the student, reintegrate them into classroom life, and at the same time avoid making mistakes suggest that teachers may experience uncertainty and feelings of inadequacy during this process. Similarly, recent studies have shown that teachers need more information and support regarding the course of the illness, the student's physical limits, appropriate classroom approaches, and possible risks.^7^^,^^39^ This suggests that school personnel should be understood not only as implementers, but also as stakeholders in need of support. The present findings indicate school-side needs for practical and understandable guidance, which could be relevant to future interdisciplinary models involving pediatric oncology nurses. Such models may consider ways to translate treatment-related information into school-useable guidance, clarify health-related precautions, and help school personnel understand possible fluctuations in energy, concentration, infection sensitivity, or emotional responses during reintegration.

The protective role of home-based education and hospital-school support as a bridge was another important aspect of the findings. However, it is important to distinguish educational continuity during treatment from full reintegration into the child's regular school. School personnel's appreciation of preserving the student's bond with school suggests that continuity of transition is important not only for academic performance, but also for sustaining belonging. Systematic reviews reporting that educational support is more effective when continuity is maintained support this finding.^4^^,^^5^ From this perspective, home-based education, hospital-school support, distance learning, or intermediate support strategies that maintain regular contact with school should be understood as bridging mechanisms rather than as substitutes for full regular school reintegration. These forms of support may reduce learning loss and help students continue to feel that they remain connected to a school community before or alongside later return to their own classroom and peer group. The accounts of hospital-school teachers mainly reflected educational continuity during treatment and transition, whereas regular school personnel's accounts reflected later readjustment to classroom routines, peer relationships, and school-based expectations in the child's own school. For nursing-informed interdisciplinary planning, this continuity may be relevant because it frames school reintegration as a gradual transition rather than an abrupt return. School personnel's accounts suggest that maintaining contact with school during treatment may create opportunities for anticipatory guidance, staged preparation, and collaborative planning before full classroom re-entry into the child's regular school setting.

A key contribution of the third analytic domain is school-side support needs and coordination gaps. The findings indicate that school personnel often try to manage the process through individual effort, goodwill, and experience; however, sustainable support requires systematic information flow, clear role definitions, and inter-institutional coordination. This is consistent with recent review and qualitative studies reporting that school reintegration support in many contexts progresses in fragmented, person-dependent, and non-standardized ways.^10^^,^^40^^,^^41^ In particular, information transferred between the health care system and the school may be limited, delayed, or detached from context, leading school personnel to experience uncertainty about how to support the student. Therefore, effective school return requires more than merely sharing the medical diagnosis; meaningful, understandable, and pedagogically useable information is needed about the student's functional capacity, possible challenges, accommodations they may need, and appropriate forms of support. This finding is especially relevant to pediatric oncology nursing as an inferred area of interdisciplinary collaboration, because the school-side accounts suggest a need for clinical information to be translated into practical guidance for day-to-day school participation. However, because the data were obtained from school personnel, this role should be interpreted as an inferred practice opportunity rather than as a direct participant demand. In future interdisciplinary return-to-school models, these school-side needs could indicate potential areas in which pediatric oncology nurses may be involved, such as identifying school-related concerns before return, supporting family preparation for communication with the school, contributing to child-specific return-to-school planning, clarifying precautions and activity limits, and maintaining follow-up communication as needs change over time.

At this point, pediatric oncology nursing represents a potential area of intersection between clinical follow-up and school reintegration. It has been emphasized that psychosocial care in childhood cancer should focus not only on the disease and its treatment, but also on the child's reintegration into everyday life and social roles.^13^ Recommendations for long-term follow-up also indicate that educational outcomes and school functioning should be included as part of routine long-term follow-up after childhood cancer.^42^ Within this framework, the school-side needs identified in this study suggest potential areas in future interdisciplinary return-to-school models, including readiness assessment, identification of concerns and information needs, family-school communication, and coordination between the health care system and the school. The development of structured tools to assess return-to-school readiness further indicates that this field is becoming increasingly visible in nursing practice.^15^ More specifically, future studies that include nurses, children, families, and school personnel could examine whether and how pediatric oncology nurses may contribute to assessing readiness for school return during follow-up visits, discussing possible school-related symptoms and functional limitations with families, helping determine what information should be shared with teachers, supporting the preparation of individualized return-to-school recommendations, and participating in communication pathways that connect the family, school, and health care team. These findings suggest that school reintegration may need to be considered as a planned, interdisciplinary component of pediatric oncology supportive care, rather than as a secondary issue left to individual effort.

In the Turkish context, this study addresses an important gap in the existing literature by making visible the perspective of school personnel. Because most existing studies have focused on the experiences of children or parents, the experiential knowledge of the school side has remained limited. Yet the findings of this study show that school personnel both recognize the student's vulnerability and may experience institutional isolation while trying to provide support. For this reason, the school-side needs identified in this study may inform the development of written information packages for school personnel, online informational sessions, individualized return-to-school plans, and pre-structured communication mechanisms among schools, families, and health care teams. Pediatric oncology nurses could potentially contribute to such models by helping develop school-oriented information materials, participating in preparatory communication before return, and supporting continuity between clinical follow-up and school reintegration planning. However, future studies that include pediatric oncology nurses, children, and families are needed to examine these potential roles directly. Recent studies showing the feasibility of community-based support initiatives for school personnel also suggest that such structured approaches may have practical value.^43^ Future research should evaluate the effects of interdisciplinary, nursing-informed, or nurse-supported return-to-school interventions on students' psychosocial adjustment, school participation, peer relationships, and teacher self-efficacy.

### Implications for nursing practice and research

Although the findings have indirect implications for pediatric oncology nursing, they highlight areas in which pediatric oncology nurses can contribute through interdisciplinary collaboration with health care professionals, educators, and families. The following implications should therefore be interpreted within the context of this collaborative role rather than as direct nursing responsibilities. Returning to school for children and adolescents undergoing or having completed childhood cancer treatment may benefit from being approached as a planned and coordinated transition rather than a process managed through individual effort alone. In practice, this could involve structured information sharing among schools, families, and health care teams, together with individualized return-to-school plans, gradual adjustment arrangements, peer preparation, and psychosocial support. These plans could be developmentally sensitive and could consider the child's age, school level, academic expectations, peer relationships, health-related vulnerabilities, and treatment-related sensitivities such as infection risk, fatigue, limited endurance, medication-related needs, concentration difficulties, visible bodily changes, and emotional vulnerability. Hospitals and health care teams may consider providing school-oriented, understandable, and practical information about the child's health-related needs, possible activity limitations, infection-related precautions, fatigue management, warning signs requiring family or health care contact, and appropriate classroom accommodations. Families may also benefit from support in communicating the child's needs, preferences, emotional concerns, and changing capacity for school participation to school personnel. For clinical care, these findings suggest that school re-entry could be considered throughout cancer treatment and follow-up as part of interdisciplinary supportive care, and that health care teams may consider preparing school-oriented information before the child returns to school rather than responding only after difficulties emerge. Based on the school-side needs identified in this study, potential areas in future interdisciplinary return-to-school models may include readiness assessment, identification of health-related and psychosocial support needs, clarification of information for school personnel, and facilitation of communication across settings. Within schools, identifying a responsible contact person, such as a teacher, counselor, administrator, or a school nurse or a nurse working in a school infirmary, may help maintain communication, monitor the child's adjustment, and revise the re-entry plan when needs change. Brief training and consultation support for school personnel may also strengthen both student adjustment and staff confidence in managing the process. Future intervention studies including children, families, school personnel, and pediatric oncology nurses are needed to examine the feasibility and effects of interdisciplinary, nursing-informed, or nurse-supported school re-entry models.

### Limitations

This study has several limitations. First, it was conducted within a specific context and with a limited number of school personnel; therefore, the transferability of the findings to other settings may be limited. Participants were recruited from different school-related settings rather than from a single school; however, the study was not designed as a multi-center comparison of school systems, institutional policies, procedures, or resources. Therefore, differences across schools and institutional contexts may have influenced participants’ experiences, but these differences could not be analyzed comparatively. Accordingly, the findings cannot be used to compare school systems or institutional policies. Although participants were recruited from different school roles and institutional settings, the number of participants in some professional subgroups was limited. In particular, only one school counselor and one school nurse were included; therefore, role-specific saturation was not claimed, and role-specific conclusions cannot be drawn for these professional groups. Recruitment and non-participation should also be considered when interpreting the sample. Of the 19 individuals approached and screened, 14 were eligible and 12 participated. Two eligible teachers could not participate because of workload and scheduling difficulties. Within the snowball recruitment route, six school nurses were screened; one eligible school nurse participated, while five were ineligible because they lacked direct experience with the school re-entry process of a child with cancer. No participant withdrew after providing consent.

Second, the data were based solely on the narratives of school personnel; the perspectives of children, parents, and health professionals, including pediatric oncology nurses, were not included in this study. Therefore, the findings reflect school-side experiences and should not be interpreted as representing the direct expectations of children, families, or health care professionals. Accordingly, the nursing-related implications of this study should be interpreted as indirect and practice-relevant inferences derived from school-side experiences, rather than as direct evidence of pediatric oncology nurses' roles, responsibilities, or expectations. Because pediatric oncology nurses were not interviewed, this study cannot directly define pediatric oncology nurses' roles in school reintegration. In addition, the study was based on school personnel's accounts rather than on a child-level case registry. Although participants working in regular school settings were from different school contexts and their accounts were treated as distinct school re-entry experiences, the exact total number of unique school re-entry cases represented across all interviews could not be determined with certainty. This was because some participants described multiple-student experiences, and possible overlap may have occurred particularly in the student experiences described by the two hospital-school teachers who worked in the same hospital-school/clinic context.

Third, although participants described experiences involving children and adolescents from different school levels, the sample was not designed to compare developmental age groups. Therefore, the findings cannot generate age-specific recommendations for elementary school children, adolescents, or older students separately. Fourth, recurrence, relapse, and death did not emerge as central themes in the school re-entry cases discussed by participants; therefore, these issues were not analyzed as separate dimensions of school reintegration. Future studies should directly examine how schools prepare for and respond to recurrence, relapse, clinical deterioration, or death in the context of childhood cancer. Finally, the interviews were conducted online, which may have limited the reflection of some contextual details. Nevertheless, the study provides an important contribution by offering a focused account of school personnel's experiences.

### Strengths

A key strength of this study is that it addresses the return-to-school process from the perspective of school personnel. This school-side perspective provides insight into how return to school is observed, interpreted, and supported within the everyday school context. Bringing together different roles, such as teachers, administrators, a school counselor, and a school nurse, enabled the academic, social, emotional, and institutional dimensions of the process to be understood in an integrated way. In addition, presenting the findings within a structured framework comprising academic and health-related adjustment, social and emotional reintegration, and school-side support needs and coordination gaps made the multilayered nature of return to school more visible. The use of two researchers and field notes supported reflexive discussion and contextual interpretation.

## Conclusions

This study showed that, from the perspective of school personnel, school re-entry is not merely the resumption of academic attendance but a multidimensional process of reintegration requiring the restoration of belonging, the reorganization of peer relationships, and institutional support. The findings underscore the importance of coordinated, individualized planning that considers developmental stage, school level, academic expectations, peer relationships, health-related needs, and effective communication among schools, families, and health care professionals to support educational continuity throughout the cancer care continuum. Although the implications for pediatric oncology nursing are indirect, the identified school-side needs suggest that pediatric oncology nurses may have a valuable collaborative role in future return-to-school models; however, this potential contribution requires direct investigation in future research.

## CRediT authorship contribution statement

Birgül Erdoğan: Conceptualization, Methodology, Investigation, Data curation, Formal analysis, Project administration, Writing – original draft, Writing – review & editing.

Tuba Arpaci: Conceptualization, Methodology, Investigation, Formal analysis, Supervision, Validation, Resources, Writing – review & editing. All authors have read and approved the final manuscript.

## Ethics statement

Ethical approval for this study was obtained from the Health Sciences Scientific Research and Publication Ethics Committee of Karamanoğlu Mehmetbey University (Approval No. 03-2025/27; March 5, 2025). Families provided verbal permission solely to facilitate contact with relevant school personnel. Children and families were not study participants, and no data were collected directly from them; therefore, formal child assent was not obtained. Written informed consent was obtained from all participating school personnel. Limited contextual information, specifically the student's age group and duration of absence from school, was reported by school personnel and recorded without direct identifiers. No student names, contact details, medical-record information, or other direct identifiers were collected, and no medical or school records were accessed. All participants were informed of the voluntary nature of participation, their right to withdraw at any time, and the measures taken to protect data confidentiality.

## Data availability statement

De-identified data may be available from the corresponding author upon reasonable request, subject to approval by the relevant ethics committee and to participant confidentiality and data-protection restrictions.

## Declaration of generative AI and AI-assisted technologies in the writing process

During revision, ChatGPT (OpenAI) was used solely for language editing and wording refinement. After using this tool, the authors reviewed and edited the content as needed and take full responsibility for the content of the publication. No generative AI or AI-assisted technology was used for data collection, analysis, interpretation, or figure creation.

## Funding

This research did not receive any specific grant from funding agencies in the public, commercial, or not-for-profit sectors.

## Declaration of competing interest

The authors declare that they have no known competing financial interests or personal relationships that could have appeared to influence the work reported in this paper.
