# Supplementary material for: School re-entry for children and adolescents with cancer: A qualitative study from the perspectives of school personnel
Source: Asia Pac J Oncol Nurs. 2026 Jul 24;13:101009. doi: 10.1016/j.apjon.2026.101009 (PMC13448483; doi:10.1016/j.apjon.2026.101009)
Supplement: Supplementary file 1 [file mmc1.docx]

Supplementary Table S1. Main domains of the semi-structured interview guide and sample questions

| **Interview domain** | **Sample question** |
| --- | --- |
| **Initial impressions regarding return to school** | How did you experience the return to school of a student who had been away from school for a long time due to cancer treatment? |
| **School-based preparation and arrangements** | Were any preparations or arrangements made within the school before the student returned? |
| **Academic adjustment** | What difficulties did you observe in terms of the student’s participation in lessons, learning process, or academic performance? |
| **Psychosocial adjustment and peer relationships** | What can you say about the student’s peer relationships, position in the classroom, and social adjustment? |
| **Communication with the family and healthcare team** | How did you experience the communication process with the family or healthcare team? In what areas did you need information? |
| **Challenges and facilitating factors** | What were the main factors that hindered or facilitated the return-to-school process? |
| **Support needs and recommendations** | What kinds of support do you think school personnel need in order to manage this process more effectively? |
