# Supplementary material for: School re-entry for children and adolescents with cancer: A qualitative study from the perspectives of school personnel
Source: Asia Pac J Oncol Nurs. 2026 Jul 24;13:101009. doi: 10.1016/j.apjon.2026.101009 (PMC13448483; doi:10.1016/j.apjon.2026.101009)
Supplement: Supplementary file 2 [file mmc2.docx]

**COREQ (Consolidated Criteria for Reporting Qualitative Research) Checklist**

| Item | Description |
| --- | --- |
| Domain 1: Research Team and Reflexivity | |
| 1. Interviewer/facilitator | All interviews were attended by two researchers. The first researcher conducted the interviews, while the second researcher observed the process and took field notes. |
| 2. Credentials | The researchers held PhD degrees in pediatric nursing. |
| 3. Occupation | The researchers were female academic researchers/faculty members in pediatric nursing with experience in pediatric oncology and qualitative research. |
| 4. Gender | Female. |
| 5. Experience and training | The researchers had experience in pediatric oncology and qualitative research. The interview guide was developed by two faculty members with PhD degrees in pediatric nursing who had research experience in pediatric oncology and qualitative research. Expert opinions were obtained from one faculty member with expertise in qualitative research and three faculty members with PhD degrees in pediatric nursing who had experience in pediatric oncology. |
| 6. Relationship established | There was no hierarchical or direct work relationship between the researchers and the participants. Participants were contacted after eligibility was established and written informed consent was obtained. |
| 7. Participant knowledge of the interviewer | Participants were informed about the aim and scope of the study, the voluntary nature of participation, their right to withdraw at any time, and confidentiality of the data. At the beginning of each interview, the researchers also explained their roles in the interview process. |
| 8. Interviewer characteristics | The researchers’ pediatric nursing background, pediatric oncology experience, and qualitative research experience were reported. Reflexive discussions were conducted throughout data collection and analysis to consider how the researchers’ professional backgrounds might influence interpretation. |
| Domain 2: Study Design | |
| 9. Methodological orientation | Qualitative descriptive design; data were analyzed using team-based inductive thematic analysis informed by Braun and Clarke’s six phases. |
| 10. Sampling | Purposive sampling, specifically maximum variation sampling, was used. Snowball sampling was applied when needed to facilitate access to information-rich participants. |
| 11. Method of approach | Families of children and adolescents who were receiving or had completed cancer treatment were initially contacted through the hospital. Children and families identified or suggested school personnel involved in educational continuity or school re-entry, and families provided verbal permission solely to facilitate contact. Before any contact details were shared, families confirmed that the suggested school personnel agreed to have their information provided to the researchers. The researchers then introduced the study and obtained written informed consent from those who agreed to participate. Children and families were not study participants, and no data were collected directly from them; therefore, formal child assent was not obtained. Hospital-school teachers were approached directly, and additional participants were identified through snowball referrals. |
| 12. Sample size | Twelve school personnel participated in the study: eight teachers, two school administrators, one school counselor, and one school/infirmary nurse. |
| 13. Non-participation | A total of 19 individuals were approached and screened through three recruitment routes. Six school personnel were approached through the family-mediated route, and all six were eligible and participated. Two hospital-school teachers were approached directly, and both participated. Eleven individuals were approached through snowball sampling; six were eligible, of whom four participated and two teachers could not be interviewed because of workload and scheduling difficulties. The remaining five individuals were school nurses who did not meet the eligibility criterion of having direct experience with the school re-entry process of a child with cancer. Overall, 14 individuals were eligible, 12 participated, two eligible individuals did not participate, and five were ineligible. No participant withdrew after providing consent. |
| 14. Setting of data collection | Data were collected online via Zoom. Online interviews were preferred because participants were located in different school or hospital-school settings and this format allowed interviews to be scheduled at times convenient for school personnel without disrupting school routines. |
| 15. Presence of non-participants | No non-participants were reported as being present during the interviews. Interviews were conducted individually at times convenient for the participants, in quiet and uninterrupted settings. |
| 16. Description of sample | Participant characteristics included professional role, institutional setting, age group, gender, education, professional-experience category, cancer-related training, and experience supporting children with cancer during school re-entry. Limited contextual information included the students’ age group/school level, duration of school interruption, and timing of the most recent school re-entry experience. No direct student identifiers were recorded. |
| 17. Interview guide | A semi-structured individual interview guide was developed by the researchers based on literature on school re-entry after childhood cancer, prolonged school absence, educational continuity, psychosocial adjustment, peer relationships, family-school-healthcare communication, and information needs during school reintegration. Expert opinions were obtained from one faculty member with expertise in qualitative research and three faculty members with PhD degrees in pediatric nursing who had experience in pediatric oncology. The guide was reviewed for clarity, relevance, comprehensiveness, and appropriateness, and the wording and flow of the questions were refined based on expert feedback. |
| 18. Repeat interviews | Each participant was interviewed once. No repeat interviews were conducted. |
| 19. Audio/visual recording | With participants’ permission, audio recordings were made. The recordings were transcribed verbatim after each interview. |
| 20. Field notes | Field notes were taken during and at the end of each interview. Emotional tone, hesitation, emphasis, and visible non-verbal cues observable through Zoom, such as facial expression and pauses, were noted when relevant. |
| 21. Duration | Each semi-structured interview lasted approximately 30–45 minutes. Completion of the Descriptive Characteristics Form took approximately 5 minutes and was conducted before the interview. |
| 22. Data saturation | Data collection and preliminary analysis were conducted concurrently. Recruitment continued until sufficient thematic depth and repetition were observed across interviews. Saturation was assessed through regular review of emerging codes, patterns, and thematic categories after each set of interviews. By the later interviews, no substantially new codes were identified and additional interviews primarily elaborated already established categories. Saturation was assessed at the level of the overall thematic structure rather than separately for each professional subgroup. |
| 23. Transcripts returned | Full transcripts were not returned to participants. However, participants reviewed the main points and field notes at the end of the same interview session, immediately after the interview, as a form of meaning validation. |
| Domain 3: Analysis and Findings | |
| 24. Number of data coders | Coding and theme development were undertaken independently by two researchers, after which the codes and themes were discussed until consensus was reached. |
| 25. Description of coding tree | The analytic structure was presented as three analytic domains, eight themes, and related subthemes. The analytic domains, themes, and subthemes are presented in Table 2. |
| 26. Derivation of themes | Themes were derived inductively from the data using Braun and Clarke’s six-phase thematic analysis. The analysis was conducted using an inductive and data-near descriptive approach focused on participants’ reported experiences, observations, concerns, and suggestions. |
| 27. Software | No qualitative data analysis software was used. Coding and theme development were conducted manually by the researchers through repeated reading, independent coding, comparison, discussion, and analytic notes. |
| 28. Participant checking | Participants reviewed the main points and field notes at the end of each interview as meaning validation. Formal participant feedback on the final themes was not reported. |
| 29. Quotations presented | Participant quotations were presented to support the findings. Quotations were identified by participant code and role, such as teacher, hospital-school teacher, school administrator, school counselor, or school/infirmary nurse. |
| 30. Data and findings consistent | Findings were grounded in participant accounts and supported by direct quotations. The researchers also reviewed whether each theme was sufficiently supported by participant quotations and whether claims in the Discussion were grounded in the Results. |
| 31. Clarity of major themes | Three analytic domains were clearly presented: school personnel’s observations of academic and health-related adjustment; school personnel’s perceptions of social and emotional reintegration; and school-side support needs and coordination gaps. |
| 32. Clarity of minor themes | Eight themes and related subthemes were presented in Table 2 and described in the Results section. Differences across participant roles and institutional settings were considered during analysis and used for contextual interpretation rather than formal subgroup comparison. |
